# Estimating Distribution of Hidden Objects with Drones: From Tennis Balls to Manatees

**DOI:** 10.1371/journal.pone.0038882

**Published:** 2012-06-25

**Authors:** Julien Martin, Holly H. Edwards, Matthew A. Burgess, H. Franklin Percival, Daniel E. Fagan, Beth E. Gardner, Joel G. Ortega-Ortiz, Peter G. Ifju, Brandon S. Evers, Thomas J. Rambo

**Affiliations:** 1 Florida Fish and Wildlife Conservation Commission, Fish and Wildlife Research Institute, St. Petersburg, Florida, United States of America; 2 Florida Cooperative Fish and Wildlife Research Unit, United States Geological Survey, Department of Wildlife Ecology and Conservation, University of Florida, Gainesville, Florida, United States of America; 3 Department of Forestry and Environmental Resources, North Carolina State University, Raleigh, North Carolina, United States of America; 4 Department of Mechanical and Aerospace Engineering, University of Florida, Gainesville, Florida, United States of America; University of Western Ontario, Canada

## Abstract

Unmanned aerial vehicles (UAV), or drones, have been used widely in military applications, but more recently civilian applications have emerged (e.g., wildlife population monitoring, traffic monitoring, law enforcement, oil and gas pipeline threat detection). UAV can have several advantages over manned aircraft for wildlife surveys, including reduced ecological footprint, increased safety, and the ability to collect high-resolution geo-referenced imagery that can document the presence of species without the use of a human observer. We illustrate how geo-referenced data collected with UAV technology in combination with recently developed statistical models can improve our ability to estimate the distribution of organisms. To demonstrate the efficacy of this methodology, we conducted an experiment in which tennis balls were used as surrogates of organisms to be surveyed. We used a UAV to collect images of an experimental field with a known number of tennis balls, each of which had a certain probability of being hidden. We then applied spatially explicit occupancy models to estimate the number of balls and created precise distribution maps. We conducted three consecutive surveys over the experimental field and estimated the total number of balls to be 328 (95%CI: 312, 348). The true number was 329 balls, but simple counts based on the UAV pictures would have led to a total maximum count of 284. The distribution of the balls in the field followed a simulated environmental gradient. We also were able to accurately estimate the relationship between the gradient and the distribution of balls. Our experiment demonstrates how this technology can be used to create precise distribution maps in which discrete regions of the study area are assigned a probability of presence of an object. Finally, we discuss the applicability and relevance of this experimental study to the case study of Florida manatee distribution at power plants.

## Introduction

**Figure 1:**
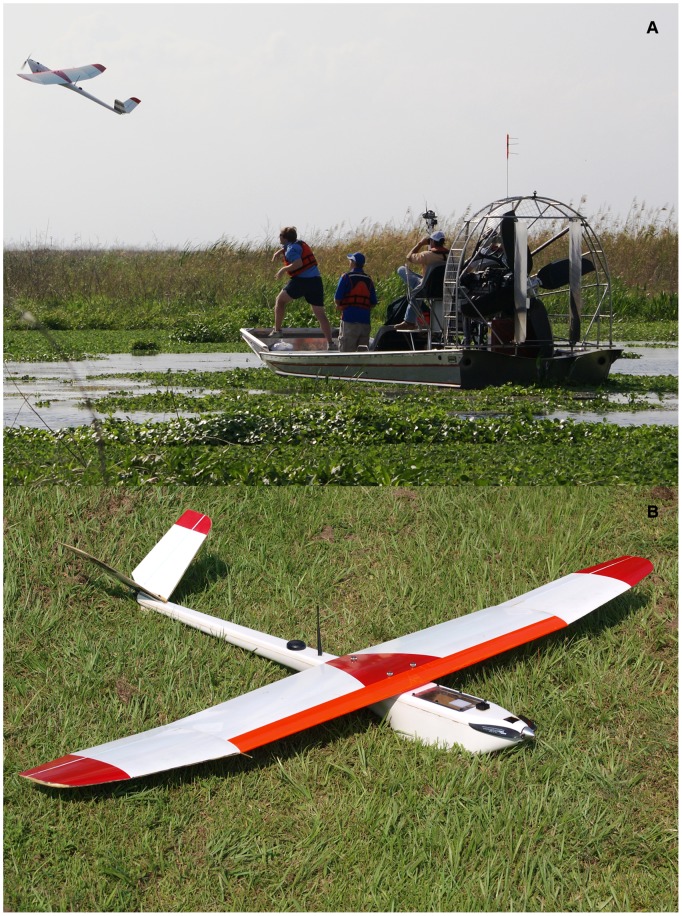
Unmanned aerial vehicle (UAV).

**Figure 2:**
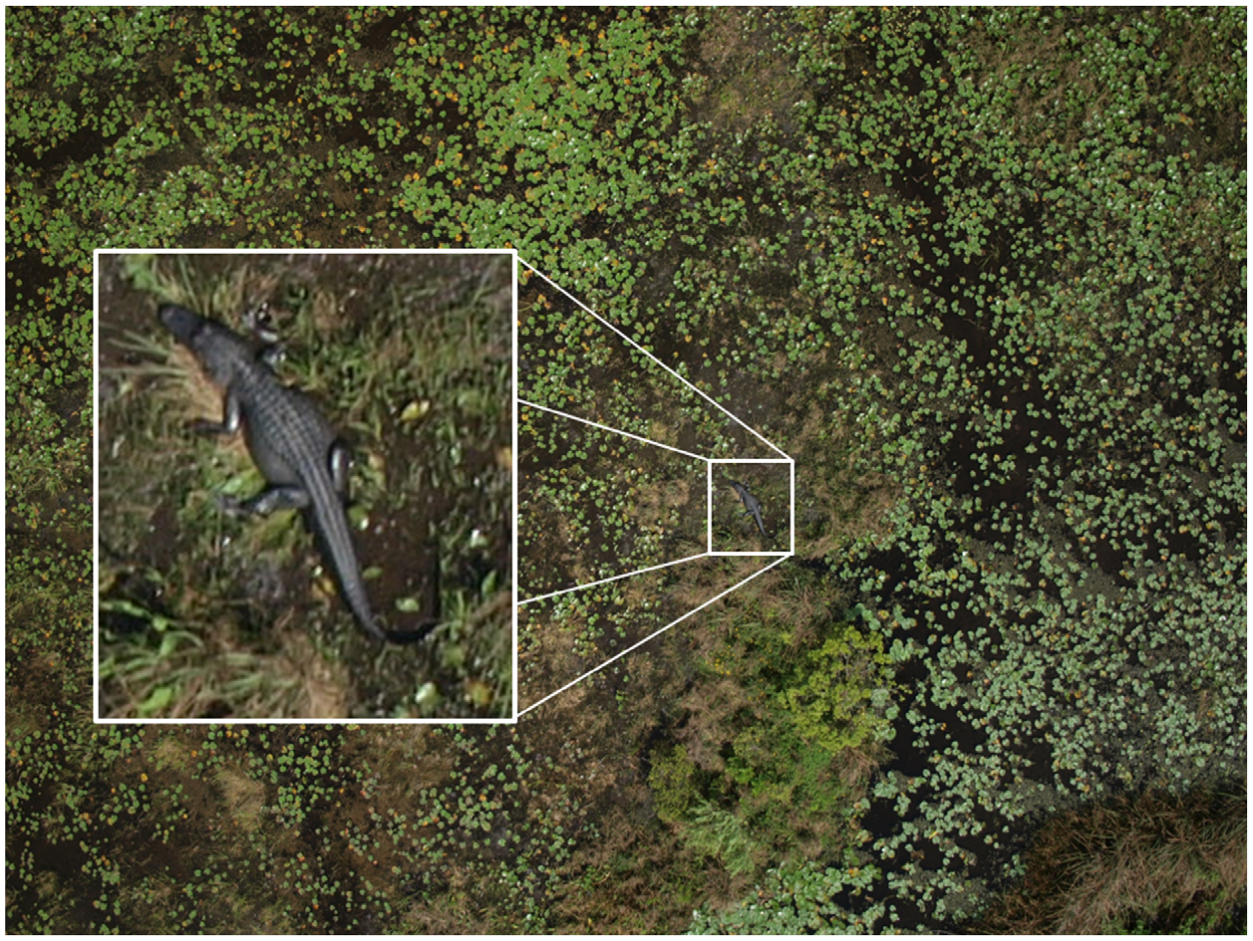
Alligator taken from the Nova UAV in Lake Okeechobee, FL. The alligator was estimated to be 1.7 m in total length. Flight altitude was 85 m.

Aircraft have long been used to conduct environmental monitoring surveys [1]–[4]. However, aircraft can be hazardous (especially when monitoring areas that are dangerous to survey, e.g., flying over forest fires), expensive, and may disturb wildlife, and the results of an aircraft survey can be misleading if an unknown proportion of the population being surveyed is not detected [5], [6]. Unmanned aerial vehicles (UAV), or drones (Figure 1), have been widely used in military applications. Recently, civilian applications have emerged, including: traffic monitoring, law enforcement, oil and gas pipeline threat detection, and the monitoring of wildlife species [1], [7], [8] (Figure 2 shows the photograph of an American alligator *Alligator mississippiensis* in Lake Okeechobee taken with a UAV). UAV may have several advantages over manned aircraft for wildlife surveys, including reduced ecological footprint, increased safety, and the ability to collect high-resolution geo-referenced imagery that can document the presence of species without the use of a human observer. We illustrate how geo-referenced data collected with UAV technology in combination with recently developed statistical models can improve our ability to estimate distribution and abundance of organisms that are temporarily undetectable and whose distribution is determined by an environmental gradient (e.g., temperature, vegetation, soil type). We demonstrate how this technology can be used to estimate abundance and distribution of organisms that are otherwise difficult to enumerate. We create precise distribution maps in which discrete regions (grid cells) of the study area are assigned a probability of presence of an object even if that object was not observed during a survey. We discuss how this method can be used to accurately describe the relationship between water temperature and manatee distribution in space.

**Figure 3:**
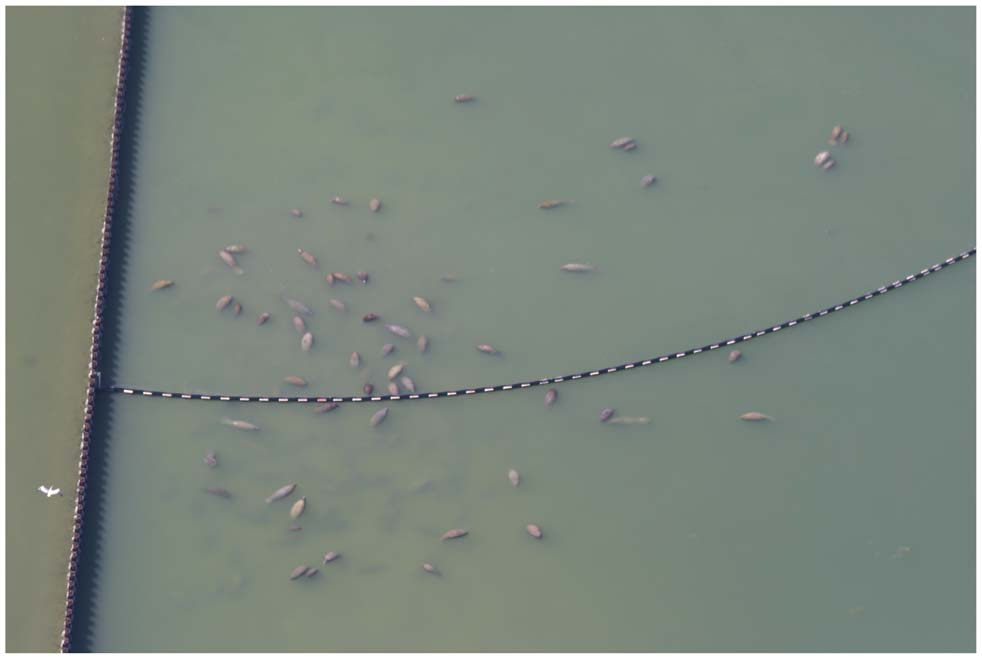
Groups of Florida manatees at a power plant in Florida during a cold event.

Our study was motivated by our work on the endangered Florida manatee, *Trichechus manatus latirostris* (Figure 3). We are interested in determining the spatial distribution of manatees in warm-water refugia, such as power plants effluents, where they aggregate in large numbers during cold weather (i.e., greater than 1,000 manatees have been counted at some power plant sites during a survey). In particular, we are interested in quantitatively evaluating the relationship between water temperature and the spatial distribution of manatees. By collecting surface-water temperatures and taking geo-referenced photographs of the site, it may be possible to elucidate the relationship between temperature and the presence of individual manatees. Knowledge of this relationship would be useful in determining warm water carrying capacity at these aggregation sites. Carrying capacity has long been a parameter of interest to biologists, ecologists, and resource managers because of its potential in assessing population dynamics of the manatee. Before implementing this approach to monitor manatees at an aggregation site, we conducted a field experiment to demonstrate the efficacy of our method. In this approach, we used tennis balls as surrogates for manatees. This approach entailed collecting images with a UAV of a 30 m × 30 m grid (each cell in the grid was 1 m × 1 m) containing a known number of tennis balls, each of which had a certain probability of being hidden or undetectable. We then applied spatially explicit occupancy models with a Bayesian approach to the data to estimate the number of balls present in the grid and to create precise distribution maps of the probability of presence of a ball in each grid cell.

## Materials and Methods

### UAV

The University of Florida UAV Research Team has been actively developing small UAV systems with the explicit use as a remote sensing platform for natural resources and ecological monitoring for the last 12 years. The electric-powered Nova 2.1 used in this study was a hand-launched, 2.7 m wingspan aircraft weighing 4.5 kg, which was hand constructed out of foam, fiberglass, carbon fiber, and Kevlar, and had a maximum sustainable airtime of approximately one hour (Figure 1A and 1B). Autonomous flight control was achieved with a Procerus® Technologies Kestrel™ 2.2 autopilot system aboard the aircraft, which was linked by a 900 MHz wireless modem to Virtual Cockpit™ 2.6 autopilot software on the ground. Pre-planned fight paths were uploaded and autonomously executed by the aircraft. The autopilot system allowed for autonomous takeoffs and landings, instantaneous flight plan changes, and a user-friendly interface. The optical payload for the Nova 2.1 consisted of a commercial-off-the-shelf 10 megapixel Olympus® E-420 digital single-lens reflex camera with a 25 mm ‘pancake’ lens. The optical payload was outfitted with its own GPS-aided Inertial Navigation System (GPS/INS) for improved direct geo-referencing. Synchronization of the camera shutter and the GPS/INS was achieved with a custom circuit board to timestamp each acquired image with a navigation data packet. Telemetry files and images generated during a flight were stored onboard the aircraft via a 1.8 GHz Microsoft Windows XP® micro form factor computer with an 80 GB solid-state hard drive. The optical payload was capable of producing images with approximately 5 cm ground resolution at 200 m flight altitude. In this study, we flew at 200 m altitude to capture the entire 900-cell experimental grid within a single image frame, while maintaining sufficient ground resolution of the tennis balls. Because the individual images captured with the Nova 2.1 UAV were directly geo-referenced, post-processing of the imagery allowed researchers to measure individual targets (e.g., length, area) within an image. This feature allows researchers to measure an animal without having to physically capture it (Figure 2).

We note that the application of UAV technology is new and still involves many restrictions (e.g., obtaining Federal Aviation Administration (FAA) permission to operate in civil airspace) [1]. However, our own experience has been that as this technology has become more widely used it also has become increasingly less difficult to operate a UAV in many areas of the National Airspace System.

### Experimental Design

**Figure 4:**
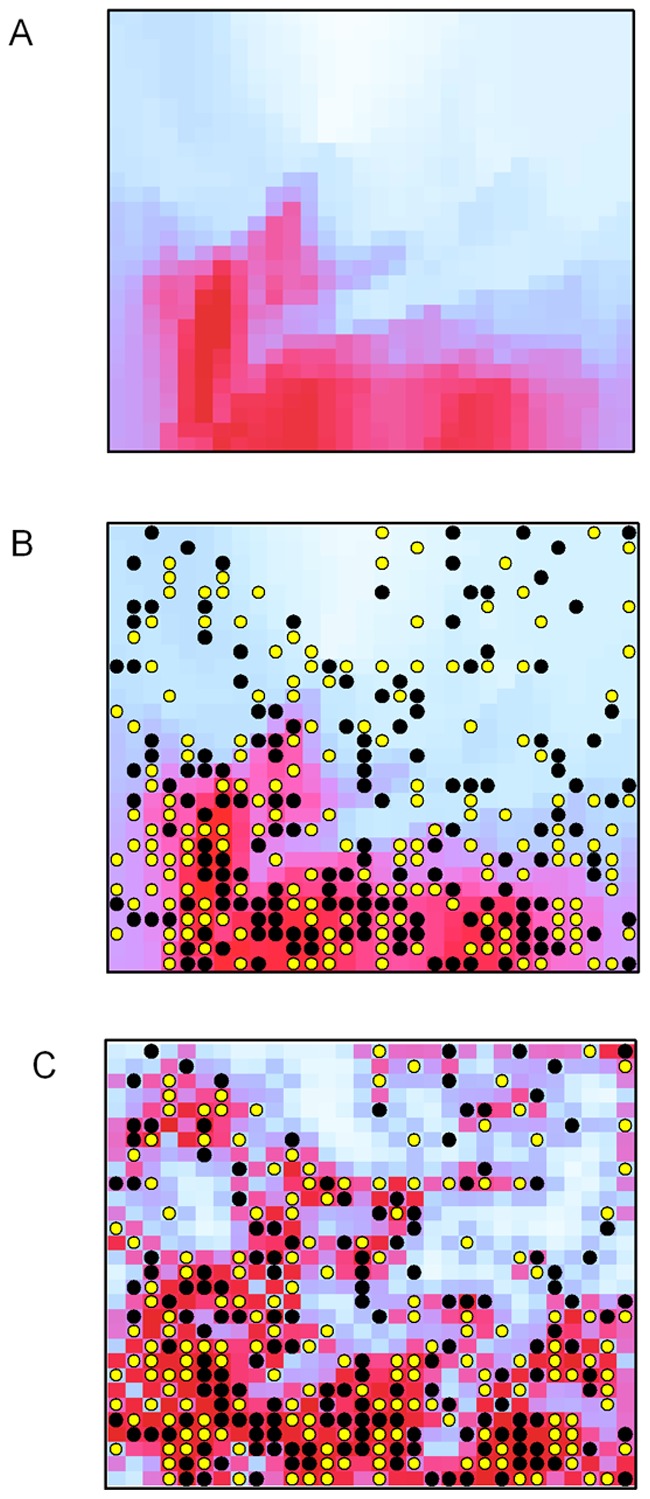
Estimates of conditional probability of occurrence as a function of temperature, and the number of neighboring sites that are occupied. 4A: Temperature gradient (light blue: lower temperatures; dark red: higher temperatures). 4B: Model 2, conditional probability of occupancy modeled as a function of temperature (light blue corresponds to lower probabilities; dark red represents higher values). 4C: Model 3, conditional probability of occurrence (spatial model). True locations of the objects are denoted by circles: yellow circles correspond to the objects available for detection on the first UAV survey, black circles correspond to the location of the hidden objects (i.e., those not available for detection).

**Figure 5:**
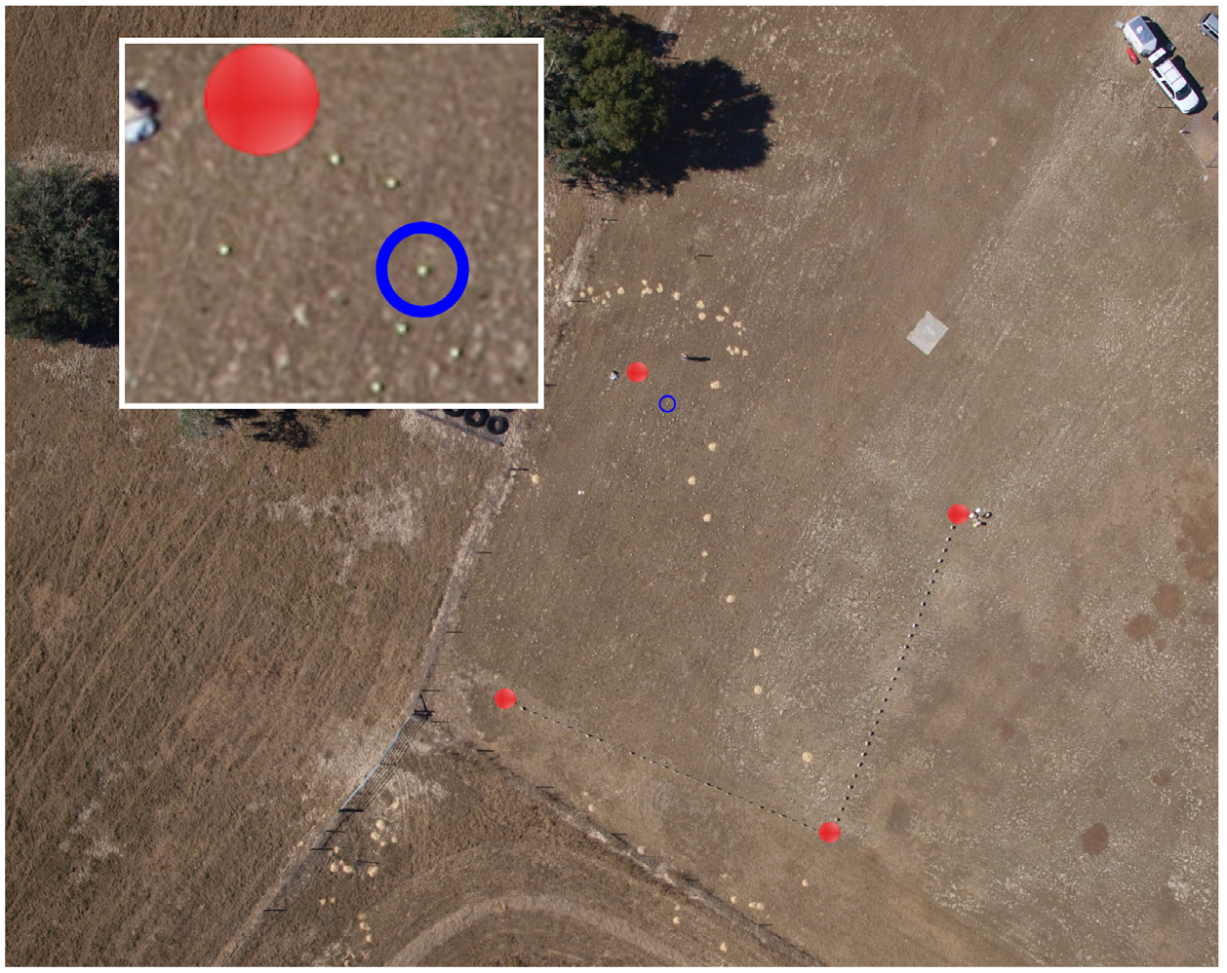
Experimental setup taken from the unmanned aerial system (UAV) at an altitude of 200 m. Red circles indicate the corners of the grid; inset shows a section of the photograph enlarged. Blue circle indicates a tennis ball available for detection.

We created a 30 m × 30 m grid (each cell was 1 m × 1 m) and placed a tennis ball in each of 329 selected cells; each ball represented the presence of an object (e.g., a manatee at a power plant; Figures 3). The spatial distribution of the balls was determined by a simulated gradient (Figure 4A). In our application on manatees, the simulated gradient was one of water temperature; however for other species it could represent other relevant ecological variables (e.g., vegetation type, soil type, elevation, salinity). Digital photographs of the balls were taken by the UAV during three passes flown over the grid at an altitude of 200 m and an air speed of 16 m/s (Figure 5). Images of the grid were taken every 2.5 s as the UAV autonomously flew pre-programmed passes. After each pass, we either covered the ball (hiding it from view) or allowed it to remain uncovered. The probability of a ball being covered during each pass was set at 0.50. A single observer, who had not helped set up the experiment, counted all the objects seen in photographs taken by the UAV during each of its passes over the grid.

### Data Analyses

We applied three occupancy models to the data:

Model 1 assumed that the probability of occupancy (ψ) was the same for all cells in the grid (i.e., it was a nonspatial model); Model 2 modeled ψ as a function of the environmental gradient; and Model 3 modeled ψ as a function of the occupancy status of the neighboring cells (i.e, an autologistic model [9]).

Each of these models accounts for imperfect detection by the observer. For the purpose of mapping, it was useful to compute the conditional probability of occurrence in cell *i*, ψ*_ci_*, which corresponded to the probability that an object was present in cell *i* given that no object was observed there during the survey (i.e., it was temporarily unavailable). We applied occupancy models [9]–[11] to simultaneously estimate the probability of occupancy of an object at cell *i* (ψ*_i_*) and the probability of detection of this object at cell *i* given that the object was present at cell *i* during survey *j* (*p_ij_*). To estimate the probability of detection at cell *i*, repeated surveys were required at each cell *i*. These models also assumed that the occupancy status within each cell did not change between surveys.

Our experimental study area was a grid composed of *R* = 900 cells. Each cell *i* had a probability ψ*_i_* of being occupied:
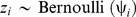
(1)where *z_i_* = 1 indicates a cell is occupied, otherwise *z_i_* = 0. We surveyed each of the *R* cells 3 times during the survey. This sampling resulted in observations, *y_ij_*, of detection or non-detection of objects at each cell *i* during each survey *j*. We assumed the observations arose from a Bernoulli distribution such that:
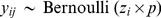
(2)where *p* is the probability of an object being detected at cell *i* during survey *j*. Under each of the models, we held *p* constant for all cells and surveys.

We modeled the potential variation in occupancy, ψ*_i_*, in three different ways. The first was a naïve model (Model 1) with occupancy detection probabilities being a constant value across all cells. The logit-linear form of the model is specified as:
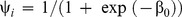
(3)


For Model 2, we modeled the probability of occupancy as a function of our simulated temperature covariate (*G_i_*), which was specified for each cell *i*.
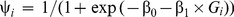
(4)


Finally, we incorporated a spatial correlation by using an auto-logistic model [9], Model 3, which specifies a relationship between each cell and its neighbors. Here, we defined the neighborhood (*M_i_*) around a cell *i* and then averaged the occupancy state (*z_m_*) of the neighbors to be incorporated as a covariate (*x_i_*). Thus, the model was specified as:
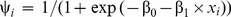
(5)where



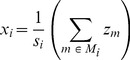
(6)Thus *x_i_* is the average occupancy state of the neighbors of cell *i*, *M_i_* is the collection of cells that are neighbors of *i*, *s_i_* is the cardinality of *M_i_*; and *z_m_* is the occupancy state of each neighbor.

**Figure 6:**
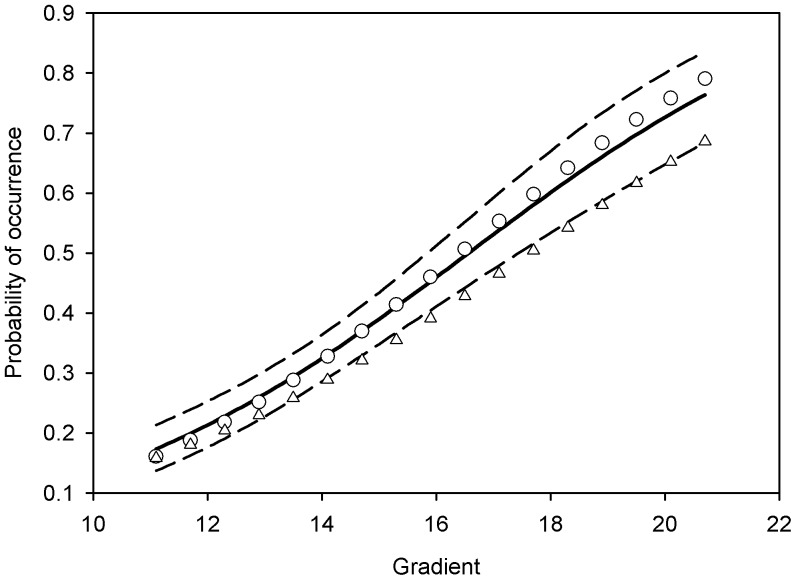
Relationship between the temperature gradient and the probability of occurrence. Solid line corresponds to the relationship between the temperature gradient and the probability of occurrence obtained from Model 2; dashed lines correspond to the 95% CI. Circles represent the relationship between the temperature gradient and the probability of occurrence based on the true locations of the balls, triangles indicate the relationship obtained from the maximum count.

When detection probability, *p* is 1, then the occupancy model can be reduced to a simple logistic regression type model. We applied a logistic regression with a Bayesian approach to the maximum count data (after the 3 surveys) and to the true location of the balls, to estimate the intercept and the slope parameter of the relationship between the gradient and the probability of occurrence (triangles and circles in Figure 6). The logistic regression approach is useful if the objects move among cells between the surveys and if detection can be assumed to be perfect.

The probability that a cell was occupied by an object given that no objects were detected after multiple *K* surveys was noted 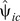 [10].

Based on the estimates of occupancy and detection from the model, 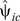 is derived as:
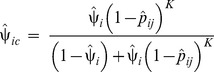
(7)


The circumflex symbol indicates that these parameters are estimates. We present 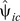 (Figure 4) to show the estimated probability of occupancy given that no objects were observed.

All data sets were analyzed with program R version 2.13. We fitted Models 1, 2, and 3 using a Bayesian approach and Markov chain Monte Carlo simulation methods [9]; these analyses were conducted with WinBUGS version 1.4 using the R package R2WinBUGS. We ran 3 parallel chains, each with 20,000 iterations, and discarded the first 15,000 iterations.

## Results and Discussion

**Figure 7:**
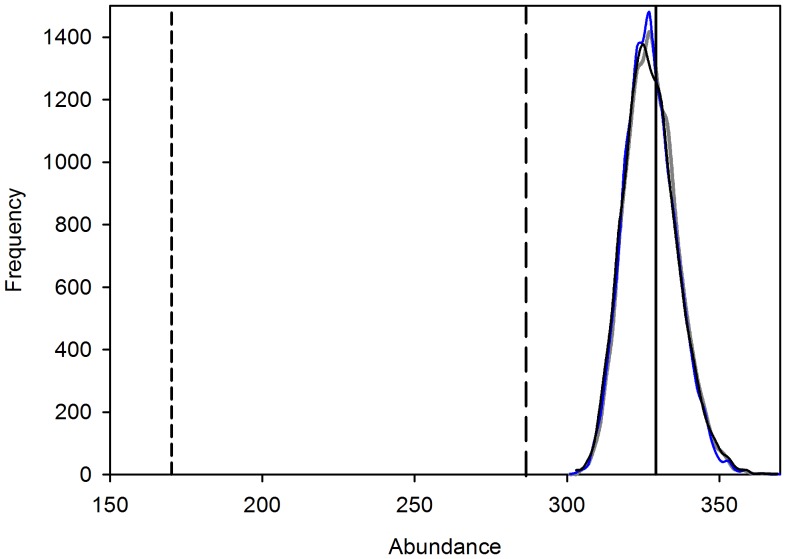
Posterior distributions of the number of occupied cells obtained from three models. Model 1 (blue curve); Model 2 (grey curve); Model 3 (black curve); true abundance of balls (solid line); maximum count of balls after three surveys of the UAV (long dashed line); count after the first survey of the UAV (short dashed line).

The estimated mean number of occupied cells (both with hidden and not hidden objects) for Model 1 (nonspatial-occupancy model) was 328 (95% CI: 313, 346, Figure 7). The estimated mean number of objects for Model 2 (occupancy modeled as a function of gradient) was 328 (95% CI 313, 347, Figure 7), and for Model 3 (occupancy modeled as a function of the number of neighbors) was 328 (95% CI: 312, 348, Figure 7). The probability of detecting an object was estimated to be 0.51 (95% CI: 0.47, 0.55) for Model 2. The count for the first pass alone was 170 objects (Figure 7). The maximum count among all 3 passes was 284 objects (Figure 7). Although these models can account for imperfect detection, they are not designed to account for over counting of objects. In this case, our observer (ball counter) failed to detect a few objects while counting from the photos, as expected (1 on the second-pass photo and 2 on the third-pass photo). What was not expected was over counting of objects that resembled the tennis balls in the photos (5 on the first-pass photo, 6 on the second-pass photo, and 2 on the third-pass photo). To reduce this problem we should have emphasized that a sighted object should not have been reported as a ball if the observer was not completely confident that it was a ball. We could also have used multiple observers. Indeed, the model that we used can correct for undercounting but not for over counting. However, in our application, there was no evidence that the over counting introduced substantial bias.

The relationship between the gradient and the probability of occurrence obtained from Model 2 and the maximum count is shown in Figure 6. The mean estimates of the probability of occurrence (solid line in Figure 6) based on Model 2 were greater than the estimates based on the maximum count (triangles in Figure 6), but were slightly lower than the true relationship at the high end of the temperature gradient (circles in Figure 6), although the true values were within the 95% credible intervals (dashed lines in Figure 6). The spatial plots of the estimates of conditional probability of occurrence for Model 2 closely match the simulated pattern for the gradient (Figures 4A and 4B). Note that a 95% CI associated with the probability of occupancy is available for each grid cell. The pattern for the conditional probability of occurrence for Model 3 also captured the gradient pattern, but not as accurately as for Model 2 (Figures 4A and 4C). Interestingly, the estimates of uncertainty obtained from the three models were similar (Figure 7). Thus, Models 2 and 3 are more informative (i.e., they provide information about spatial distribution whereas Model 1 does not) at almost no cost in terms of precision (note that Model 2 and 3 have 3 parameters, whereas Model 1 has 2 parameters). Model 2 is most useful if some environmental variable (e.g., temperature) that drives the distribution of a target species is available. If such environmental variable is not available Model 3 can still be used to determine the spatial distribution (by using the number of neighboring sites that are occupied as a covariate). This type of model may also be of particular interest in the case of species that tend to aggregate (e.g., species that demonstrate “flocking” behavior).The results of our experiment suggest that UAV are an effective means of counting objects (or species) located in areas that are difficult to survey. In this experiment, objects as small as a tennis ball were accurately detected in photographs taken by an UAV at 200 m altitude. Another benefit of this technology is that the size of the objects can be measured. In ecological applications, this feature can be useful in distinguishing young from adults (e.g., in obtaining age-specific estimates of density), if there is a discernible age-specific difference in size; or in differentiating among individuals (e.g., using scar patterns on manatees). Although the use of UAV technology in the monitoring of wildlife has been described elsewhere [7], to our knowledge the use of geo-referenced images obtained from a UAV in tandem with the application of statistical models that account for imperfect detection has not been evaluated for determining the precise distribution of hidden objects. In addition to being able to obtain accurate estimates of occurrence (here the sum of occupied cells can be used as an approximation of abundance of objects), this approach can help us understand how a species’ distribution is affected by environmental gradients (e.g., water temperature, elevation, or vegetation cover). In this case, relying on a simple count would have led to 170 tennis balls. In the case of the manatee, we can determine, based on water temperature and population density, the carrying capacity of warm-water aggregation sites, such as power plant discharges, natural springs, and other passive-thermal basins. Our experiment showed that in some cases this technology also may allow biologists to assess density and age-specific distribution as a function of the gradient because adult and young animals may, in some species, be differentiated by size. These methods could also be used to examine changes in the use of a particular site as it is related to shifts in environmental conditions (e.g., how changes in temperature or the volume of warm water at a site affect the number of manatees using that site). We demonstrated that precise measurements of an environmental gradient could lead to precise estimation of the predicted distribution of the objects in question. However, even in the absence of environmental gradient measurements, we were able to use the observed distribution of objects (with Model 3) to predict the probability of occurrence of objects even in locations where the objects were not observed. This could in fact be an interesting application for environmental monitoring. For example, given the distribution of objects and some information about the relationship between that distribution and the environmental gradient, it should be possible to determine the configuration of the gradient itself. In other words, information about the distribution of the objects (e.g., manatees) could be used to infer the spatial characteristics of the environmental gradient (e.g., temperature; see similarities between Figures 4A and 4C).

Our simulation demonstrates the efficacy of using UAV technology in combination with statistical models to understand and describe relationships between organisms that are difficult to detect, and environmental factors that influence distribution of such species. Using these methods, not only can accurate estimates of distribution be obtained, but the relationship between the distribution of the individuals and the environment can also be better estimated. It is worth pointing out a few limitations and potential for future improvement. Firstly, in our experiment the probability of detecting balls was 0.5 at each site, in many situations detection probability can vary in space and time. If such variation is to be expected, it is possible to account for this variation in the models. For instance, if visibility is affected by water clarity or wave actions, detection probability could be modeled as a function of these visibility covariates [9] [11]. Temporal variation in detection can also be accounted for in the models [9], [10]. Secondly, the occupancy model that we used in our analysis assumed closure, i.e., the occupancy status in each cell did not change among pictures taken by the UAV. Short surveys (e.g., that are minutes apart) can help meet this assumption. In the case of species that move extensively, one option is to use spatially explicit capture mark recapture models that keep track of individuals (e.g., for instance with manatees individuals may be distinguished by their color, shape size, and scar patterns). If animals move extensively but cannot be individually identified, but detection probability is excellent (e.g., manatees aggregated at a warm water site with clear water), the logistic regression approach that we presented can still be applied to determine the relationship between the temperature gradient and manatee distribution. Finally, most models to estimate abundance and distribution of animals from repeated count data, do not account for overcounting, which can lead to bias. Accounting for this type of error in these models is an active area of research. We anticipate that the simultaneous progress in model developments and UAV technology will lead to improvement in the accuracy of environmental monitoring. Therefore, it is important for users of this technology to remain informed about the latest developments in both UAV engineering and statistical modeling.

In conclusion, we believe that the methods that we described have great potential for environmental monitoring, especially for species that are difficult to observe or that live in areas that are difficult for a human observer to adequately monitor at the faster speeds and higher altitudes flown by most standard manned-survey aircraft. Additionally, these methods also could be useful for other types of environmental monitoring, especially in areas that are dangerous to survey, such as mapping and detection of forest fires (e.g., smoke detection), and oil spill monitoring. We expect to continue to use data collected by UAV in combination with other environmental variables in monitoring manatees, and hope that other applications will be developed to improve conservation and monitoring of other wildlife and plant species.
